# Evaluation of the Antimicrobial Efficacy of *N*-Acetyl-l-Cysteine, Rhamnolipids, and Usnic Acid—Novel Approaches to Fight Food-Borne Pathogens

**DOI:** 10.3390/ijms222111307

**Published:** 2021-10-20

**Authors:** Ondrej Chlumsky, Heidi J. Smith, Albert E. Parker, Kristen Brileya, James N. Wilking, Sabina Purkrtova, Hana Michova, Pavel Ulbrich, Jitka Viktorova, Katerina Demnerova

**Affiliations:** 1 Department of Biochemistry and Microbiology, University of Chemistry and Technology, Technicka 5, 166 28 Prague 6, Czech Republic; Sabina.Purkrtova@vscht.cz (S.P.); Hana.Michova@vscht.cz (H.M.); pavel.ulbrich@vscht.cz (P.U.); jitka.viktorova@vscht.cz (J.V.); demnerok@vscht.cz (K.D.); 2 Center for Biofilm Engineering, Montana State University, Bozeman, MT 59717, USA; heidi.smith@montana.edu (H.J.S.); albert.parker@montana.edu (A.E.P.); kristen.brileya@montana.edu (K.B.); james.wilking@montana.edu (J.N.W.); 3 Department of Microbiology and Cell Biology, Montana State University, Bozeman, MT 59717, USA; 4 Department of Mathematical Sciences, Montana State University, Bozeman, MT 59717, USA; 5 Chemical and Biological Engineering Department, Montana State University, Bozeman, MT 59717, USA

**Keywords:** *N*-Acetyl-l-cysteine, rhamnolipids, usnic acid, bacterial growth, biofilm, antimicrobial efficacy, food-borne pathogens, minimum inhibitory concentrations, acute cytotoxicity

## Abstract

In the food industry, the increasing antimicrobial resistance of food-borne pathogens to conventional sanitizers poses the risk of food contamination and a decrease in product quality and safety. Therefore, we explored alternative antimicrobials *N*-Acetyl-l-cysteine (NAC), rhamnolipids (RLs), and usnic acid (UA) as a novel approach to prevent biofilm formation and reduce existing biofilms formed by important food-borne pathogens (three strains of *Salmonella enterica* and two strains of *Escherichia coli*, *Listeria monocytogenes*, *Staphylococcus aureus*). Their effectiveness was evaluated by determining minimum inhibitory concentrations needed for inhibition of bacterial growth, biofilm formation, metabolic activity, and biofilm reduction. Transmission electron microscopy and confocal scanning laser microscopy followed by image analysis were used to visualize and quantify the impact of tested substances on both planktonic and biofilm-associated cells. The in vitro cytotoxicity of the substances was determined as a half-maximal inhibitory concentration in five different cell lines. The results indicate relatively low cytotoxic effects of NAC in comparison to RLs and UA. In addition, NAC inhibited bacterial growth for all strains, while RLs showed overall lower inhibition and UA inhibited only the growth of Gram-positive bacteria. Even though tested substances did not remove the biofilms, NAC represents a promising tool in biofilm prevention.

## 1. Introduction

Food-borne pathogens are responsible for infections and intoxications with significant effects on human health and adverse economic impacts for the food industry worldwide [1,2,3]. Contamination may occur at any stage during food processing as a result of insufficient cooking during food preparation, improper food storage, unhygienic food handling, inadequate refrigeration, or cross-contamination from surfaces in direct contact with food or directly from infected people [1,4]. Food industries create nutrient-rich environments, and their insufficient sanitation may be conducive to microbial growth and persistence, which facilitates long-term colonization and continuous contamination of the product [5]. To effectively combat biofilms in the food industry, efficient and frequent biofilm eradication methods are needed [6,7].

Conventional sanitization strategies in the food industry rely on physical and chemical actions through the use of antimicrobials, sanitizers, or disinfectants to remove biofilm and inhibit the growth of biofilm-associated microorganisms. Biofilms, clusters of microorganisms that usually stick to surfaces, are dynamic, highly organized communities of organisms embedded in extracellular polymeric substances (EPS), such as exopolysaccharides, extracellular DNA (eDNA), proteins, and lipids, and are highly resistant to conventional eradication methods [8,9,10,11]. Due to slow penetration through the biofilm, cells attached in the deeper layers have a longer time to adapt to adverse conditions. Furthermore, their physical proximity and the presence of eDNA result in horizontal gene transfer and further spread of antimicrobial resistance [12,13,14]. This strongly suggests that additional, potentially less conventional approaches are needed to control biofilm formation in food industry settings. One possible strategy is to employ natural substances, such as *N*-Acetyl-l-cysteine (NAC), rhamnolipids (RLs; R90), and (+)-usnic acid (UA), all of which previously showed promising antimicrobial efficacy against important clinical pathogens [15,16,17,18,19,20,21,22,23,24,25,26,27,28,29,30,31,32,33]. Other benefits of these substances are their solubility, affordability, and chemical stability. Therefore, they have high potential for effective biofilm control in food processing plants, both for prevention of biofilm formation and reduction in existing biofilms. However, little is known about their cytotoxicity and antimicrobial efficacy against food-borne pathogens frequently occurring in the food industry.

NAC is readily soluble in water and is routinely applied in medical treatments to combat cystic fibrosis, chronic bronchitis, cancer, paracetamol intoxication, and others [15,16]. NAC is a derivative of the amino acid L-cysteine and is well-known as a potent thiol-containing antioxidant and mucolytic agent that disrupts disulfide bonds in bacterial proteins [16,17]. In addition, NAC plays a key role in the breakdown of the extracellular polymeric substances (EPS) and thus prevents biofilm maturation and diminishes cell protection [18]. Although the antimicrobial efficacy has been demonstrated in several studies [18,19], little is known about the antimicrobial properties of NAC on important food-borne pathogens, except for anti-biofilm activity against *E. coli* and methicillin-resistant *S. aureus*. Regarding the cytotoxicity, it has been reported that NAC induced apoptosis of H9c2 cells; however, in other studies, it served as a cytoprotective compound [20,21].

RLs are water-soluble biosurfactants that are defined as glycolipids composed of one or two rhamnose molecules linked to one or two fatty acid alkyl chains [22]. While RLs can be produced by diverse organisms [23,24,25,26], the best-studied ones were isolated from cultures of *Pseudomonas aeruginosa* [23,26]. They have been utilized in a wide range of applications, including medicine (anticancer and antibiofilm agents), cosmetics (cosmetic formulations), the food industry (additives and antimicrobial agents), biofuels, and in bioremediation of pesticides and heavy metals [24,25,26,27]. Although the mechanism of action is not completely understood, it is supposed that RLs may disturb membrane structure through interactions with phospholipids and membrane proteins [23,28]. RLs selected for this study are declared as non-toxic. However, the cytotoxic effect may be changed by alteration of RLs chemical structures [24]. Regarding the antimicrobial effect of RLs on planktonic cells and biofilms, RLs can reduce cell adhesion, affect biofilm maturation, and disrupt mature biofilms, especially of Gram-positive bacteria [24,25,28]. However, the anti-biofilm activity has been tested only against methicillin-resistant *S. aureus* and *L. monocytogenes* [24,25].

UA demonstrates low solubility in water but high solubility in organic solvents. It is a dibenzofuran derivative naturally occurring in several lichen species and has been previously used in medical treatments (oral care), pharmaceuticals (topic ointments), and cosmetics (cosmetic formulations) [29,30]. The antimicrobial activity of UA is primarily caused by inhibition of DNA and RNA synthesis [31]. However, some evidence suggests that UA is only able to inhibit bacterial growth (*S. aureus* and *E. coli*) and prevent biofilm formation of Gram-positive bacteria [29,30,31,32]. UA was effective at inducing some alterations in the morphology of Gram-negative biofilms indicating that UA interfered with signaling pathways. Nevertheless, planktonic cells remained resistant to UA treatment [29,32]. Although UA is potentially hepatotoxic [29,30,32], low concentrations of UA did not cause damage to the liver [30].

In this study, four significant food-borne pathogens of relevance to the food industry and public health (three strains of *Salmonella enterica* and two strains of *Escherichia coli*, *Listeria monocytogenes*, *Staphylococcus aureus*) were selected to test the antimicrobial efficacy of NAC, RLs, and UA. The effectiveness of each natural substance was evaluated by determining minimum inhibitory concentrations needed for inhibition of bacterial growth, biofilm formation, metabolic activity, and biofilm reduction. Modern microscopy techniques, such as transmission electron microscopy (TEM) and confocal scanning laser microscopy (CLSM), distinguishing live and membrane-compromised cells in combination with quantitative image analysis using Beer’s Law to model the attenuation of the laser were used to visualize and quantify the impact of tested substances on cells in both planktonic and biofilm phases of growth. The possible acute cytotoxicity of individual substances was verified in vitro to determine their applicability in the food industry.

## 2. Results

Ten concentrations of NAC, RLs, and UA were tested to determine the minimum inhibitory concentration for planktonic growth, and subsequently, the six highest concentrations were applied on preformed biofilms to evaluate the biofilm eradication ability of the compounds. MIC was defined as the lowest substance concentration able to inhibit at least 80% of microbial growth (MICPC_80_ for planktonic cells, MICBC_80_ for further growth of biofilm cells), inhibit 80% of metabolic activity (MICBM_80_ for biofilm metabolic activity, MICMPB_80_ for metabolic activity of preformed biofilm), prevent biofilm formation by at least 80% (MICBF_80_ for biofilm formation), or reduce a preformed biofilm by at least 80% (MICBR_80_ for biofilm reduction). The results of MICs are summarized in Table 1 (planktonic cells) and Table 2 (biofilm-associated cells).

### 2.1. The Effect of N-Acetyl-l-Cysteine

The interactions of NAC with bacteria were independent of their Gram-positive or Gram-negative characteristics, as the application of NAC resulted in complete inhibition of planktonic cell growth, metabolic activity, and the prevention of biofilm formation of all tested bacteria (Table 1). For preformed biofilms, NAC was able to inhibit further growth of biofilm cells and inhibit its metabolic activity for all tested strains (Table 2). Additionally, NAC reduced the mass of preformed biofilm in most of the tested strains (Table 2), except for *S*. *aureus* 1241, *L. monocytogenes* 164, and *E. coli* 683/17 (reduction of 31, 46, and 68%, respectively).

### 2.2. The Effect of Rhamnolipids

Unlike NAC, the inhibition of planktonic growth was not observed in any tested strain. The MICPC_80_ could be determined only for *L. monocytogenes* 149; for other strains, the MICPC_80_ is conceivably greater than the maximal tested concentration (1000 µg/mL), which yielded a maximum inhibition of 49–75% for Gram-positive and 23–50% for Gram-negative bacteria (Table 1). RLs were additionally able to prevent biofilm formation, except for *S*. Enteritidis ATCC 13076, where the maximum achieved inhibition for MICBF_80_ was 16% (Table 1). The MICBM_80_ for biofilm metabolic activity was determined for all tested strains (Table 1). RLs were shown to inhibit further growth of biofilm cells and the metabolic activity of preformed biofilms for both Gram-positive and Gram-negative bacteria (Table 2). Moreover, the biofilm biomass was reduced for all tested strains (Table 2).

### 2.3. The Effect of Usnic Acid

The effect of UA on tested bacteria was strongly dependent on the cell wall characteristics. For Gram-positive bacteria, UA was able to inhibit the planktonic growth and prevent biofilm formation and biofilm metabolic activity (Table 1), as well as to inhibit further growth of biofilm cells and their metabolic activity (Table 2). The only exception was *S*. *aureus* 816, where the MICMPB_80_ could not be determined as the maximal inhibition was 67%. On the other hand, for Gram-negative bacteria MICPC_80,_ MICBF_80,_ and MICBM_80_ could not be determined as the inhibitory concentration was higher than the maximal tested concentration (31.3 µg/mL, Table 1), which gave a maximum inhibition of 20–42% (MICPC_80_), 10–47% (MICBF_80_), and 16–47% (MICBM_80_). For preformed biofilms of Gram-negative bacteria, the maximum observed inhibition reached up to 61% for further growth of biofilm cells and up to 53% for its metabolic activity (Table 2). Regarding biofilm reduction, UA was only able to reduce preformed biofilm of *L. monocytogenes* 164. In other strains, the maximum observed reduction was 35–72% for Gram-positive and up to 30% for Gram-negative bacteria (Table 2).

### 2.4. Transmission Electron Microscopy Imaging

**Figure 1:**
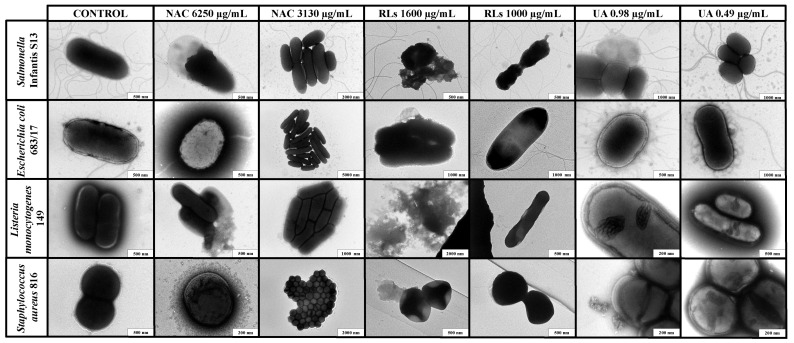
Interactions of tested substances *N*-Acetyl-l-cysteine (NAC), rhamnolipids (RLs), and usnic acid (UA) with planktonic cells of *Salmonella* Infantis S13, *Escherichia coli* 683/17, *Listeria monocytogenes* 149, and *Staphylococcus aureus* 816 visualized by TEM after 8 h of exposure.

To confirm the efficacy of tested substances on bacterial growth inhibition, selected bacterial strains were treated with effective concentrations of natural substances (NAC, RLs, and UA), and the results of individual treatments on morphology were visualized by TEM (Figure 1). The effects were compared with non-treated planktonic cells. The application of NAC minimum inhibitory concentration (6250 µg/mL) resulted in cell wall disruption and leakage of intracellular components for both Gram-positive and Gram-negative bacteria. Interestingly, a subinhibitory concentration (3130 µg/mL) resulted in the formation of clusters of increased cell division without significant changes in the appearance of the cells.

For RLs, TEM imaging confirmed a low efficacy for bacterial growth inhibition for all tested strains when the concentration of 1000 µg/mL was tested. Only after application of an increased concentration (1600 µg/mL) of RLs, we observed bacterial wall disruption and leakage of intracellular content for both Gram-positive and Gram-negative bacteria.

Visualizations of UA treatment showed some effect on DNA, suggesting that the inhibitory concentrations (0.49–0.98 µg/mL) for Gram-positive bacteria may have triggered inhibition of DNA and RNA synthesis, thus resulting in bacterial growth disruption. When the same concentrations were applied to Gram-negative bacteria, no significant effects were observed.

### 2.5. Confocal Scanning Laser Microscopy Analysis

Biofilm prevention and removal analyses were performed using previously determined minimum inhibitory and subinhibitory concentrations for metabolic activity (MICBM_80_, subCBM_80_; MICMPB_80_, subCMPB_80_) of NAC, RLs, and UA specific for each tested strain. Selected concentrations were used to evaluate the inhibition of biofilm formation (biofilm prevention) and the reduction in previously formed biofilms (biofilm removal) for the same set of microorganisms, with the exception of *S.* Infantis, which was excluded from the analysis due to its inability to form biofilm in the selected microtiter plate. Biofilm prevention and removal were observed using CLSM with specific staining to distinguish between live and membrane-compromised cells. Images were analyzed to evaluate (i). the total volume of the biomass, (ii). ratios of live and membrane-compromised cells, and (iii). to calculate biofilm volume reduction for individual organisms after the respective treatment types. The total biofilm volume was normalized by the area of the well and expressed as an average biofilm thickness. Complete data are provided in Supplementary Materials (Tables S1–S8).

#### 2.5.1. Biofilm Prevention

**Figure 2:**
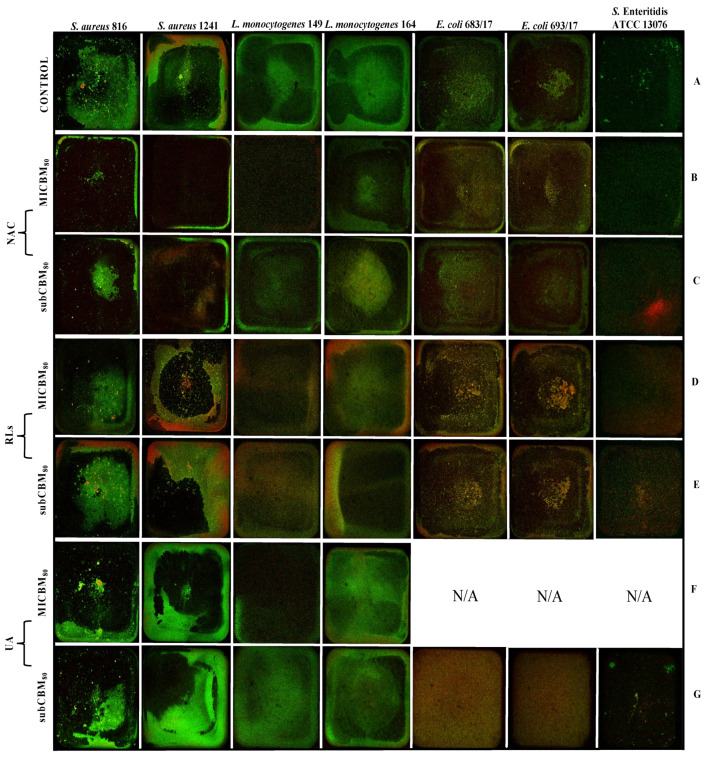
Representative projections of biofilm mass at the bottom of the microtiter wells for *Staphylococcus aureus* 816 and 1241, *Listeria monocytogenes* 149 and 164, *E. coli* 683/17 and 693/17, and *Salmonella* Enteritidis ATCC 13076 after 24 h of treatment with the respective antimicrobial compounds. (**A**): positive control; (**B**,**C**): *N*-Acetyl-l-cysteine (NAC); (**D**,**E**): rhamnolipids (RLs); (**F**,**G**): usnic acid (UA). Prior to imaging, microtiter wells were stained with LIVE/DEAD^®^ BacLight™ Bacterial Viability Kit distinguishing live cells (green) and membrane-compromised cells (red). Images represent the surface of the well 7.4 × 7.4 mm^2^. For some strains, the minimum inhibitory and subinhibitory concentrations could not be established; thus, they were not involved in the analysis. N/A marks concentration was not applied.

After 24 h of incubation, bacterial suspensions with selected compounds were visualized for biomass formation using live/membrane-compromised cell staining and CLSM. Visually, biomass images were diversified across the bacterial species but consistent within the quadruplicates for each organism. Non-treated bacteria formed biofilm predominantly localized at the edges of the wells and/or with separated dispersed biomass in the central part of the well (Figure 2A). MIC of NAC treatments mostly formed a thin layer of biomass around the edges, except for *L. monocytogenes* 164 (Figure 2B), while application of subinhibitory concentration resulted in the same trend but with the production of biomass structures similar to the non-treated biofilms (Figure 2C). Both MIC and subinhibitory concentration of RLs resulted in the production of mostly similar biomass formation as the non-treated controls (Figure 2D,E). However, propidium iodide (PI) staining revealed a higher proportion of membrane-compromised cells (*p* < 0.05), especially in both strains of *S. aureus*, *E. coli*, *L. monocytogenes* 149, and *S.* Enteritidis ATCC 13076. *S. aureus* and *L. monocytogenes* 164 biofilms treated with MIC and the subinhibitory concentration of UA demonstrated similar biomass structures as in the non-treated biofilms. In addition, the subinhibitory concentration of UA exhibited evenly distributed biomass for both *E. coli* biofilms containing membrane-compromised cells across the well, and *S*. Enteritidis ATCC 13076 demonstrated similar dispersed biomass as in the non-treated well (Figure 2F,G).

**Figure 3:**
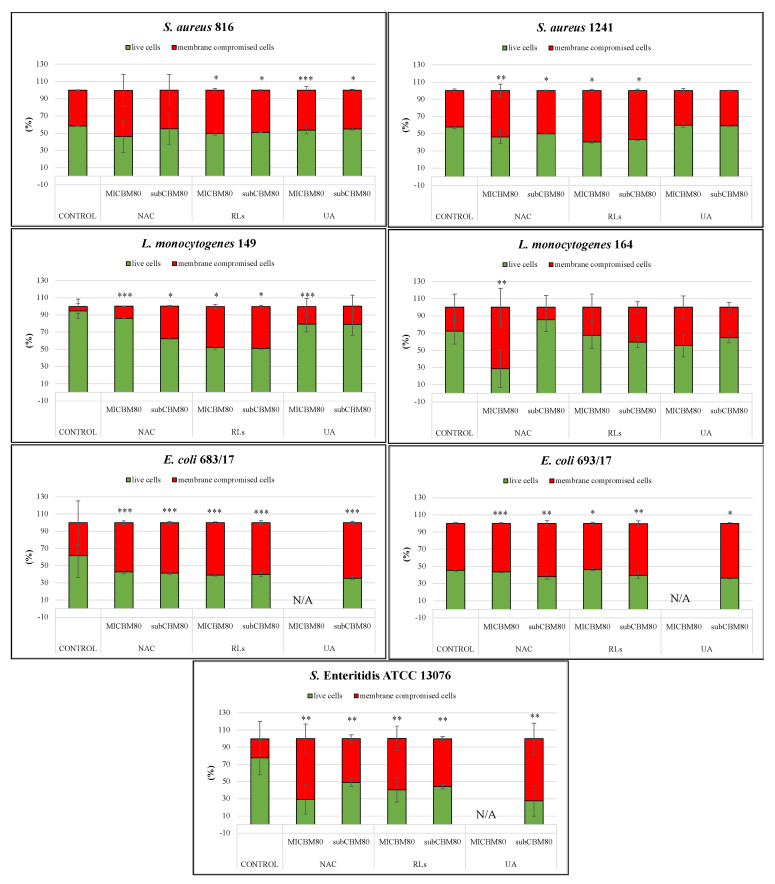
Biofilm Prevention: Ratios of live (green) and membrane−compromised cells (red) of individual organisms (*Staphylococcus aureus* 816, 1241, *Listeria monocytogenes* 149, 164, *E. coli* 683/17, 693/17, and *Salmonella* Enteritidis ATCC 13076) after 24 h of treatment with *N*-Acetyl-l-cysteine (NAC), rhamnolipids (RLs), and usnic acid (UA). Columns represent the mean and standard deviation of at least four independent technical replicates. For some strains, the minimum inhibitory and subinhibitory concentrations could not be established; thus, they were not involved in the analysis. N/A marks concentration was not applied. * marks *p* < 0.001, ** marks *p* < 0.01, *** marks *p* < 0.05, which are statistically significant differences in proportions of membrane−compromised cells comparing to the control.

In addition to visual interpretation, CLSM images were also quantitively analyzed to determine biofilm thickness and the proportion of live/membrane-compromised biomass. In total, 39 biofilms treated with MIC and subinhibitory concentrations (14 biofilms for each of the three tested compounds, except for MIC of UA, in which 11 biofilms were treated) were evaluated to establish the efficacy on the prevention of biofilm formation. Since the minimum inhibitory concentration could not be established for some strains, they were not included in the analysis. Statistically significant differences were established for 15 treated biofilms (with *p* value < 0.05 when compared to the control). However, only six cases exhibited statistically significantly thinner biofilms, whereas the rest demonstrated the opposite trend (Table 3). Interestingly, while the statistical significance of the results seemed more or less random for individual strains and corresponding treatments, *E. coli* 683/17 formed significantly thicker biofilms in all tested cases. Further, quantification showed statistically significant differences (*p* < 0.05 when compared to the control) in the proportion of membrane-compromised cells from the control (Figure 3, Tables S1–S4). Biomass viability from treated *E. coli* and *S.* Enteritidis ATCC 13076 biofilms were both statistically different (*p* < 0.05 when compared to the control, Table S4) in comparison to the control for the three tested compounds at two different concentrations. For these biofilms, the proportion of membrane-compromised cells reached roughly 50% at its maximum. The results for other species of biofilms were all strain-, compound-, and concentration-dependent. The most distinctive statistical difference was observed for *S. aureus* 1241 when treated with MIC and subinhibitory concentration of NAC (*p* < 0.01 and *p* < 0.001) and *L. monocytogenes* 149 (*p* < 0.05 and *p* < 0.001), where the proportion of membrane-compromised cells increased up to 25%. Additionally, when *L. monocytogenes* 164 (*p* < 0.01) were treated with MIC of NAC, the proportion of membrane-compromised cells increased by about 45%. The proportion of membrane-compromised cells was up to 17% for both strains of *S. aureus* (*p* < 0.001) and *L. monocytogenes* 149 (*p* < 0.001) when treated with MIC and subinhibitory concentration of RLs. The proportion of membrane-compromised cells was similarly low in comparison (up to 15%) for *S. aureus* 816 when treated with MIC and subinhibitory concentration of UA (*p* < 0.05 and *p* < 0.001) and *L. monocytogenes* 149 when treated with MIC of UA (*p* < 0.05).

#### 2.5.2. Biofilm Removal

**Figure 4:**
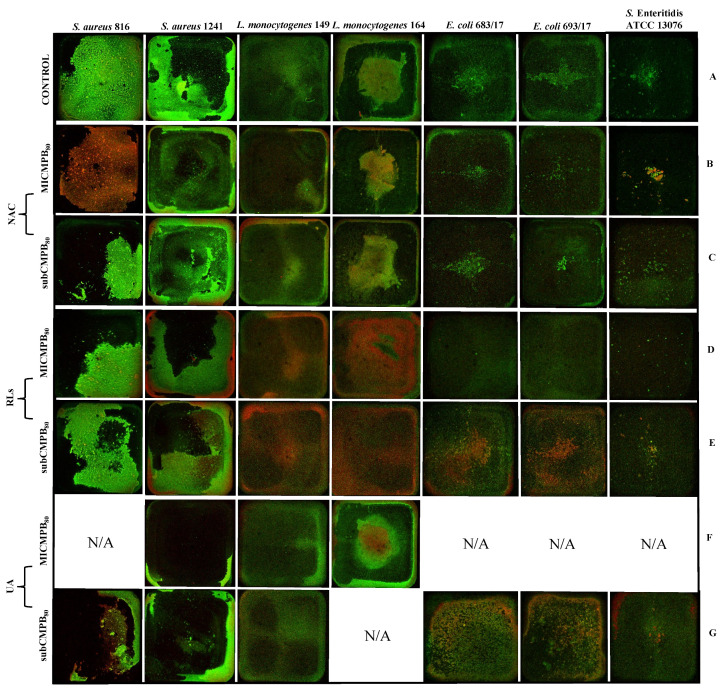
Representative projections of biofilm mass at the bottom of the microtiter wells for *Staphylococcus aureus* 816 and 1241, *Listeria monocytogenes* 149 and 164, *E. coli* 683/17 and 693/17, and *Salmonella* Enteritidis ATCC 13076 after 20 h of treatment with the respective antimicrobial compounds. (**A**): positive control; (**B**,**C**): *N*-Acetyl-l-cysteine (NAC); (**D**,**E**): rhamnolipids (RLs); (**F**,**G**): usnic acid (UA). Prior to imaging, microtiter wells were stained with LIVE/DEAD^®^ BacLight™ Bacterial Viability Kit distinguishing live cells (green) and membrane-compromised cells (red). Images represent the surface of the well 7.4 × 7.4 mm^2^. For some strains, the minimum inhibitory and subinhibitory concentrations could not be established; thus, they were not involved in the analysis. N/A marks concentration was not applied.

At first, bacterial suspensions were cultivated in microtiter plates to produce overnight biofilms (18 h). Sessile cells were then washed out, and the remaining biofilms were further cultivated for an additional 20 h with selected compounds. Following biofilm cultivation, biomass was observed using CLSM with live/membrane-compromised cell staining. Biomass images from biofilm removal treatments were similar to results from biofilm prevention, where biomass was highly diversified across the bacterial species and strains. Visually, biomass images were fairly consistent within the quadruplicates for each tested organism. Non-treated biofilms of *S. aureus* contained consistent biomass spread across the well (*S. aureus* 816) or grown around the edges (*S. aureus* 1241). The non-treated *L. monocytogenes* 149 biofilm demonstrated inconsistent biomass coverage across the well. Biomass grown around the edges with separated biomass in the central part of the well was observed for both *L. monocytogenes* 164 and both *E. coli* strains, and the non-treated *S*. Enteritidis ATCC 13076 was biomass distributed in the central part of the well (Figure 4A). MIC and subinhibitory concentration of NAC visually reduced the biomass for almost all tested strains while maintaining original pre-treatment biofilm structures. PI staining identified membrane-compromised cells spread across *S. aureus* 816 biomass and incorporated in the edge of biomass and/or in the central part of the well (Figure 4B,C). Biofilms treated with MIC and subinhibitory concentration of RLs exhibited reduced biomass but with a similar structure as in the non-treated biofilms. Membrane-compromised cells appeared contained around the edges (*S. aureus* 1241), disturbed across the well (in both strains of *L. monocytogenes*), or in separated biomass in the central part of the well (in both strains of *E. coli* when treated with the subinhibitory concentration of RLs) (Figure 4D,E). Biofilms treated with MIC and subinhibitory concentration of UA demonstrated disturbed biomass containing membrane-compromised cells across the well (in both strains of *E. coli*) or persisted biomass at the edges (in both strains of *S. aureus*, *L. monocytogenes* 149, and *S*. Enteritidis ATCC 13076) and in the central part of the well containing membrane-compromised cells (*L. monocytogenes* 164) (Figure 4F,G).

**Figure 5:**
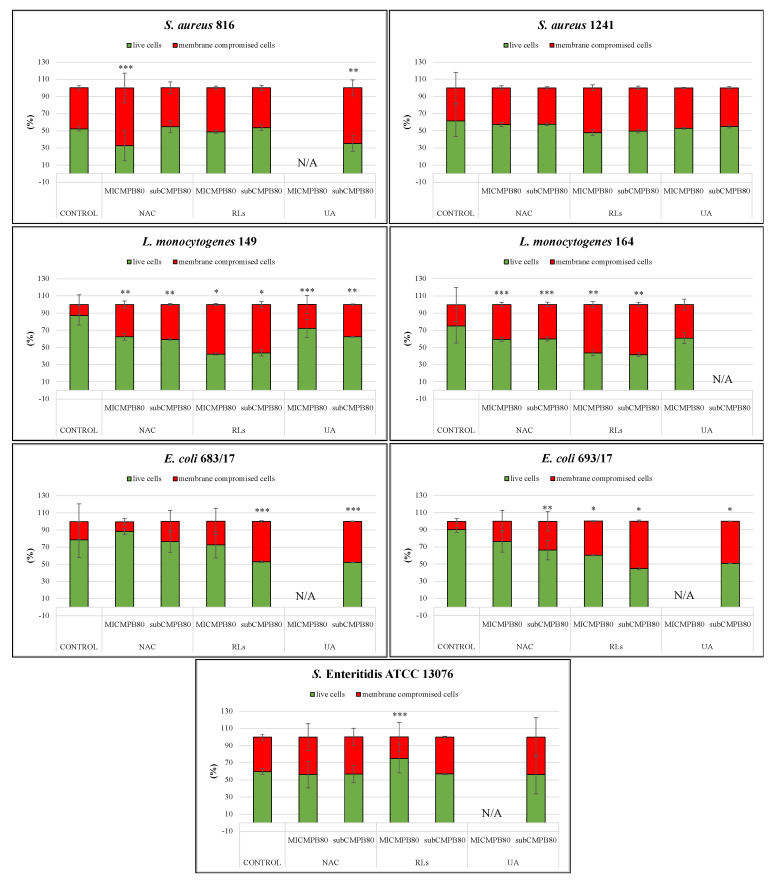
Biofilm Removal: Ratios of live (green) and membrane−compromised cells (red) of individual organisms (*Staphylococcus aureus* 816, 1241, *Listeria monocytogenes* 149, 164, *E. coli* 683/17, 693/17, and *Salmonella* Enteritidis ATCC 13076) after 20 h of treatment with *N*-Acetyl-l-cysteine (NAC), rhamnolipids (RLs), and usnic acid (UA). Columns represent the mean and standard deviation of at least four independent technical replicates. For some strains, the minimum inhibitory and subinhibitory concentrations could not be established; thus, they were not involved in the analysis. N/A marks concentration was not applied. * marks *p* < 0.001, ** marks *p* < 0.01, *** marks *p* < 0.05, which are statistically significant differences in proportion of membrane−compromised cells comparing to the control.

Similar to biofilm prevention, CLSM images for biofilm removal were further analyzed to quantify biofilm thickness and the proportion of live/membrane-compromised cells after 20 h treatment. In total, 37 biofilms treated with MIC and subinhibitory concentrations of the three tested compounds (14 biofilms for each of the three tested compounds, except for MIC and subinhibitory concentrations of UA, which treated only nine biofilms) were evaluated to establish the efficacy of the removal of preformed biofilm. Since the minimum inhibitory and subinhibitory concentrations could not be established for some strains, they were not included in the analysis. Statistically significant differences were detected for 13 of the treated biofilms (with *p* < 0.05 when compared to the control). However, only four cases exhibited significantly thinner biofilms, while the remaining nine cases exhibited thicker biomass (Table 4). Further quantification of live and membrane-compromised cells showed statistically significant differences (*p* < 0.05 when compared to the control) in the proportion of membrane-compromised cells from the control for all tested biofilms, except for *S. aureus* 1241 (Figure 5, Tables S5–S8). Out of all of the tested organisms, only the *L. monocytogenes* 149 treated biofilm demonstrated a statistically significant difference in thickness (*p* < 0.05, Table S8) for all three tested compounds at the two different concentrations, the proportion of membrane-compromised cells increased up to 45%. Differences in thickness for the other treated biofilms were strain-, compound-, and concentration-dependent. The proportion of membrane-compromised cells increased up to 20% in *L. monocytogenes* 164 when treated with MIC and the subinhibitory concentration of NAC (*p* < 0.05) and in *S. aureus* 816 when treated with MIC of NAC. Further, when treated with MIC and the subinhibitory concentration of RLs in *L. monocytogenes* 164 (*p* < 0.01) and *E. coli* 693/17 (*p* < 0.001) and the subinhibitory concentration of RLs in *E. coli* 683/17 (*p* < 0.05), the proportion of membrane-compromised cells increased up to 45%. A different finding was observed in *S.* Enteritidis ATCC 13076 when treated with MIC of RLs (*p* < 0.05), where the proportion of membrane-compromised cells was less of an increase by about 15%. Finally, treatment with the subinhibitory concentration of UA in *S. aureus* 816 (*p* < 0.01) and in both *E. coli* biofilms (*p* < 0.05 and *p* < 0.001) increased the proportion of membrane-compromised cells up to 40%.

### 2.6. Acute Cytotoxicity of Individual Natural Substances

The cytotoxic effect of natural substances (NAC, RLs, and UA) was evaluated on cell lines HRTEC, HaCat, HDF, HEK 293, HCAEC by resazurin assay over 72 h to determine the concentration of natural substances that halved the cellular viability (IC_50_, Table 5). The results indicate relatively low toxic effects of NAC (IC_50_ 460–4020 µg/mL), and higher toxic effects of RLs (IC_50_ 27.5–56.95 µg/mL), and UA (IC_50_ 0.03–4.35 µg/mL).

## 3. Discussion

In this work, three types of natural substances (NAC, RLs, and UA) were tested for their ability to inhibit bacterial growth, prevent biofilm formation, and reduce biofilm mass of four selected bacterial food-borne pathogens (Gram-positive *L*. *monocytogenes*, *S*. *aureus*, and Gram-negative *E*. *coli*, *S. enterica* Infantis, and Enteritidis). Information regarding the antimicrobial efficacy of NAC, RLs, and UA against food-borne pathogens is sparse; however, all the substances previously showed promising antimicrobial efficacy against other pathogens [15,16,17,18,19,22,23,24,25,26,27,28,29,30,31,32,33].

The efficacy of NAC has been earlier mentioned for the inhibition of biofilm formation, disruption of preformed biofilms (both initial and mature), and reduction in bacterial viability in biofilms of clinical pathogens, such as *Haemophilus influenza, Pseudomonas aeruginosa,* and *Streptococcus pneumoniae* [15,16,19]. Interestingly, these studies demonstrated lower MIC effectiveness against bacterial growth (500–2500 µg/mL) and needed for biofilm reduction (2500–10,000 µg/mL) than MIC evaluated in our study (3130–12,500 µg/mL for bacterial growth and 12,500–100,000 µg/mL for biofilm reduction). Li et al. [18] described that NAC penetrates the bacterial cell membrane and increases oxidative stress. The efficacy on bacterial growth was confirmed by TEM when the use of NAC resulted in the cell wall disruption and leakage of intracellular content for both Gram-positive and Gram-negative bacteria. In addition, NAC also interferes with the proteins and DNA in the EPS, which leads to the disruption of mature biofilm [18]. The application of MIC of NAC should have inhibited the biofilm metabolic activity of all tested strains; however, according to a more sensitive technique, CLSM, a thin biofilm consisting of live cells formed around the edges of the wells. Furthermore, CLSM did not confirm sufficient reduction in preformed biofilm. According to our results, the anti-biofilm activity is concentration- and strain-dependent. Therefore, further testing is required to validate its antibiofilm properties and effective concentration against a broad range of food-borne pathogens. Besides MICs, IC_50_ values for the cytotoxic effect of NAC were 10–100 times lower than the cytotoxicity values for RLs and UA. Although MICs for bacterial growth were mostly higher in comparison to the IC_50_ values for cell lines, the application of NAC could be still desirable if used as an additional sanitizer to common physical and chemical actions in the food industry.

On the contrary, RLs did not show any significant effect on the growth of the tested strains, despite previously reported effectivity against Gram-positive bacteria [24,25,28]. Earlier studies indicated that the growth of Gram-negative bacteria is not influenced by the presence of RLs in the media [22,28]. This may be explained by the different chemical compositions of the cell wall structures [23]. It has been suggested that the membrane of Gram-negative bacteria is hardly permeable to hydrophobic and amphipathic molecules [33]. Even though we did not achieve to determine MICs for our tested bacteria, the inhibition effect was indeed significantly higher for the Gram-positive (49–75%) than for the Gram-negative strains (23–50%). TEM detected the cell wall disruption and leakage of intracellular content for almost all tested strains. In addition, crystal violet staining and MTT assay indicated an inhibition effect on biofilm formation (MIC 15.65–250 µg/mL) and its metabolic activity (MIC 62.5–1000 µg/mL). Although CLSM analysis did not confirm reduced cell attachment, biofilms treated with RLs contained a higher proportion of membrane-compromised cells in almost all tested bacteria. This finding is contradictory with a previous study focused on RLs effect on important clinical pathogens (*P. aeruginosa,* methicillin-resistant *S. aureus*, and *Serratia marcescens*) [24], where the efficient inhibition of biofilm formation was in a lower concentration range (MIC 10–50 µg/mL). Moreover, IC_50_ values in comparison to MIC for biofilm growth demonstrated the cytotoxic effect for all tested cell lines. The cytotoxic effect of RLs mixture R90 used in our study was comparable with the findings of Aleksic et al. [24], despite the fact that this mixture is usually considered non-toxic. This could be an obstacle for the potential application in the food industry. However, the cytotoxic effect may be decreased by alteration of RLs chemical structures [24]. Finally, the last tested compound, UA, is insoluble in water but, according to previously published literature, can be dissolved in polar solvents, such as acetone, DMSO, ethanol, and ethyl acetate [30,31,34,35]. From those, DMSO is usually well tolerated with no observable cytotoxic effects at low concentrations up to 1% (*v*/*v*), followed by increasing cytotoxic effects with higher concentrations [36], leading to the inhibition of bacterial growth at 12.5% and higher [34]. In our study, the concentration range of the tested UA was prepared as two-fold dilution series by mixing the substance in 12.5% DMSO and culture medium (BHI or TSB + 1% Glc) in a 1:1 ratio. The antimicrobial efficacy of UA was observed only against Gram-positive strains. This finding is comparable with numerous studies [29,30,31,32]. Interestingly, Francolini et al. [29] determined higher minimal inhibition concentration (32 µg/mL) for *S. aureus* 1945^GFPuvr^, which inhibited bacterial growth but did not prevent the initial cell attachment. Similar findings were observed for both strains of *S. aureus* in our study but at a lower MIC for bacterial growth (0.24 µg/mL). In addition, according to the crystal violet assay, the same low concentration prevented biofilm formation. However, CLSM analysis did not confirm the notable efficacy of biofilm prevention. This could be explained by the lower adhesivity of EPS formed in the presence of UA as different microtiter plates were used for crystal violet staining (polystyrene) and CLSM analysis (ibidi Polymer). Maciąg-Dorszyńska et al. [31] demonstrated that UA at low concentrations causes rapid and strong inhibition of RNA and DNA synthesis in Gram-positive bacteria, while it did not inhibit the production of macromolecules (DNA, RNA, and proteins) in Gram-negative bacteria, which were resistant even to high doses. TEM images further confirmed the efficacy of UA on the destruction of DNA in Gram-positive bacteria, which resulted in inhibition of bacterial. Even though CLSM analysis also detected the efficacy of UA on biofilms of Gram-negative bacteria, the increased numbers of membrane-compromised cells were possibly caused by DMSO in which was UA dissolved. Regarding the cytotoxic effect, three out of five cell lines exhibited IC_50_ values higher than MICs for bacterial growth of Gram-positive strains. However, low efficacy against Gram-negative bacteria represents a huge complication for the food industry. Thus, the future application of UA is not very likely.

According to Galie et al. [5], the efficacy of natural substances on biofilm removal is sparse even with the use of other well-known substances. For this reason, their main application should be focused on the prevention of biofilm formation. In addition, some tested substances with specific strains resulted in the opposite trend when the biofilm increased when compared to the controls. A similar effect was observed in the study of Ferrer et al. [37] when subinhibitory concentrations of selected antibiotics stimulated biofilm growth in comparison to the controls.

Even though NAC is not sufficient as a tool for biofilm removal, results obtained in this study show that NAC could be used as a novel approach to prevent biofilm formation in the food industry. The application of NAC in currently employed sanitization strategies would help inhibit the growth of the biofilm-associated microorganisms that may be resistant to the conventional use of antimicrobials, sanitizers, or disinfectants.

## 4. Materials and Methods

### 4.1. Chemicals and Reagents

The liquid media used for the cultivation of bacteria were Brain Heart Infusion (BHI) or Tryptone Soya Broth supplemented with 1% glucose (TSB + 1% Glc). The following solid medium non-selective Tryptic Soy Agar (TSA) was used. All media were purchased from Oxoid (Hampshire, United Kingdom) or VWR (Radnor, PA, USA). Dimethyl sulfoxide (DMSO), 96% ethanol, and glucose were purchased from Fisher Scientific (Portsmouth, NH, USA). The chemicals 3-(4,5-dimethyl-2-thiazolyl)-2,5-diphenyl-2H tetrazolium bromide (MTT), crystal violet, sodium dodecyl sulfate (SDS), and glycerol 99% were purchased from Sigma-Aldrich (Prague, Czech Republic and Burlington, MA, USA). *N*-Acetyl-l-cysteine, rhamnolipids (R90), and (+)− usnic acid were purchased from Sigma-Aldrich (Prague, Czech Republic). Phosphate buffer solution (PBS) was bought from Lonza (Kourim, Czech Republic). The washing solution was prepared by mixing 40% DMSO solution, 1× PBS and dissolving SDS to a final concentration of 160 mg/mL_._ Dulbecco’s modified Eagle’s medium (DMEM), trypsin/EDTA solution, an antibiotic mixture (penicillin and streptomycin), fetal bovine serum (FBS), and resazurin sodium salt were purchased from Merck (Kenilworth, NJ, USA). ProxUp Basal medium with ProxUp supplements was purchased from Evercyte (Vienna, Austria). A vascular cell basal medium and an endothelial cell growth kit were purchased from ATCC (Manassas, VI, USA).

### 4.2. Bacterial Strains

All bacterial strains and their origin are listed in Table 6. They were stored at −80 °C in 25% glycerol (Sigma-Aldrich, Prague, Czech Republic) with 75% Brain Heart Infusion (Oxoid, Hampshire, United Kingdom).

### 4.3. Inoculum Preparation

Isolates were refreshed from a deep-frozen aliquot by inoculating one loopful on the non-selective Tryptic Soy Agar (TSA) purchased from the VWR (Radnor, PA, USA). Starting inoculum was prepared according to Chlumsky et al. [38]. Briefly, a single colony from an agar plate was inoculated into 2 mL of BHI and incubated at 37 °C overnight. To obtain the starting cultures, strains of *S. aureus*, *L. monocytogenes*, *E. coli,* and *Salmonella enterica* Enteritidis were centrifuged (6000× *g*, 10 min), and the resulting pellet was resuspended in 2 mL of TSB + 1% Glc, which was previously shown as an optimal medium for their biofilm formation [39]. For *Salmonella enterica* Infantis strains, the overnight grown culture was used directly as the starting culture since the same medium (BHI) was used for inoculum preparation [39]. In all cases, the inoculum was prepared by mixing chosen fresh medium for biofilm formation with the starting culture to reach a bacterial suspension of A_625nm_ ranging from 0.08 to 0.1.

### 4.4. Preparation of Natural Substances Stock Solutions and Dilution Series

Both *N*-Acetyl-l-cysteine (Sigma-Aldrich, Prague, Czech Republic) and rhamnolipids (R90; Sigma-Aldrich, Prague, Czech Republic) were dissolved in sterile distilled water to a final concentration of 100,000 µg/mL (for NAC) and 2000 µg/mL (for RLs). (+)-Usnic acid (Sigma-Aldrich, Prague, Czech Republic) was dissolved in 12.5% DMSO (Fisher Scientific, Portsmouth, NH, USA) to a final concentration of 62.5 µg/mL. Dilution series of all tested antimicrobial substances were prepared by diluting the substances in an appropriate culture medium (BHI, or TSB + 1% Glc) in a 1:1 ratio to obtain 10 different concentrations. The maximal concentrations were given by the default purity and solubility of the substances. The concentration range was 100–100,000 µg/mL for NAC, 1.96–2000 µg/mL for RLs, and 0.06–62.5 µg/mL for UA. The highest available concentrations of all three tested substances were used only for the biofilm reduction testing, where they were directly applied to a preformed biofilm. The highest tested concentration of UA (62.5 µg/mL) was excluded from further analyses due to an inhibition effect of 12.5% DMSO in which UA was dissolved.

### 4.5. Determination of Minimum Inhibitory Concentrations

The determination of MICs was performed according to Chlumsky et al. [38]. Briefly, 96-well flat-bottomed microtiter plates were inoculated with 75 µL (for biofilm formation) or 100 µL (for preformed biofilm) of bacterial suspension (A_625nm_ 0.08 to 0.1) in technical triplicates and were then carefully mixed with the dilution series of the test substances (24 h cultivation at 25 °C for *S.* Infantis, 37 °C for other strains) or were directly cultivated to preform biofilms (18 h). For a positive control of bacterial growth, the inoculum was mixed with a pure sterile medium. Furthermore, the sterile medium was included in the plate as a marker of potential microbial contamination. To evaluate planktonic cells growth (MICPC_80_) and further growth of biofilm cells (MICBC_80_), the optical density of the content of the microtiter plates was measured spectrophotometrically at 625 nm before and after 24 h (for biofilm formation) of cultivation or before and after additional 20 h incubation with the tested compound (for preformed biofilm). The difference of A_625nm_ was considered as a measure of the ability of cells to grow in the presence of the tested substances. To evaluate the inhibition of biofilm formation (MICBF_80_) and reduction in preformed biofilms (MICBR_80_)_,_ biomass was quantified using crystal violet staining [39], and its metabolic activity (MICBM_80_ and MICMPB_80_) was estimated by using the MTT (thiazyl tetrazolium bromide, Sigma-Aldrich, USA) reduction assay [39], both measured spectrophotometrically at 595 nm.

All inhibitions were calculated using the formula of Chlumsky et al. [38] below (Equation (1)):

(1) $$A_{625\left(595\right)\mathrm{nm}\;}\mathrm{inhibition}\;\left(\%\right)=\frac{A_{625\left(595\right)\mathrm{nm}}\;\left(\mathrm{control}\right)\;–{\;A}_{625\left(595\right)\mathrm{nm}}\left(\mathrm{natural}\;\mathrm{substance}\right)}{A_{625\left(595\right)\mathrm{nm}}\;\left(\mathrm{control}\right)}\times \;100$$

where $A_{625\left(595\right)\mathrm{nm}}\;\left(\mathrm{control}\right)\;$ is the absorbance of bacterial suspension/biofilm itself, and $A_{625\left(595\right)\mathrm{nm}}\left(\mathrm{natural}\;\mathrm{substance}\right)$ is the absorbance of the bacterial suspension/biofilm with the added natural substance (NAC, RLs, and UA).

The MICs represent the minimum concentrations that resulted in at least 80% inhibition of growth (MICPC_80_, MICBC_80_), metabolism (MICBM_80,_ MICMPB_80_), and biofilm formation (MICBF_80_) or resulted in at least 80% reduction in preformed biofilms (MICBR_80_). When the minimum inhibitory concentration could not be determined, the MIC was established as >the highest tested concentration for NAC, RLs, and UA.

### 4.6. Transmission Electron Microscopy Imaging

The interactions between tested natural substances and planktonic cells were visualized by TEM. The volume 0.75 mL of inoculum (A_625nm_ 0.08 to 0.1) was added into a 2 mL centrifuge tube and mixed with 0.75 mL of a natural compound of the selected concentration (the effective concentration that resulted in bacterial leakage and the concentration that guaranteed an option to observe the interaction of the tested substance with both Gram-negative and Gram-positive bacteria), or with 0.75 mL of the sterile medium (control). After cultivation (37 °C, 8 h) in a shaking incubator, a drop of a bacterial culture suspension was deposited on a copper carbon-coated electron microscopic grid and incubated at room temperature for about 10 min. After that, the excess liquid was removed by filter paper, and the grid was quickly rinsed with distilled water. Then the grid was deposited onto a solution of 1% sodium silicotungstate pH 7.4 and negatively stained for about 10 s. After the staining, the grid was left to dry and was subsequently inserted into TEM column JEOL JEM-1010 (JEOL Ltd., Tokyo, Japan), operated at 80 kV at various magnifications. Images were recorded by SIS Megaview III CCD camera and analyzed by AnalySIS v. 3.2 software (Olympus Soft Imaging Systems, Münster, Germany).

### 4.7. Confocal Laser Scanning Microscopy Analysis

#### 4.7.1. Strains and Growth Conditions

For both inhibition and reduction of biofilms, overnight cultures from BHI were diluted in TSB + 1% Glc to reach an absorbance of 0.08 to 0.1 at 625 nm. The inoculum (150 µL) was transferred into wells of the 96-well flat-bottomed Ibidiμ-Plate (Ibidi, Gräfelfing, Germany) and mixed with 150 µL of each of the three tested substances in technical quadruplicates, followed by incubation at 37 °C for 24 h. Simultaneously, the prepared bacterial suspensions were inoculated to an additional microtiter plate (200 µL) and allowed to form biofilm overnight (18 h at 37 °C). After the incubation, the content of the well was drained off, and 200 µL of each of the three tested substances was added to the plate, which was then incubated for an additional 20 h. The used concentrations of the substances represented the subinhibitory and minimum inhibitory concentrations for metabolic activity (subCBM_80_, MICBM_80_; subCMPB_80_, MICMPB_80_). Both setups contained a positive control of the bacterial growth (inoculum mixed with sterile TSB + 1% Glc medium) and a control monitoring microbial contamination (sterile medium). Non-treated wells were used as a positive control for comparison between non-treated and treated biofilm mass.

#### 4.7.2. CLSM Imaging and Image Processing Analysis

Prior to the imaging, the wells were stained with the LIVE/DEAD^®^ BacLight™ Bacterial Viability Kit (Thermo Fisher Scientific, MA, USA) following the manufacturer’s guidelines and Drago et al. [40]. Briefly, equal volumes of SYTO^®^ 9 and propidium iodide were combined with the sterile distilled water to reach a final concentration of 2.6 M. The biofilm mass was stained using 300 µL of the staining solution per well followed by incubation at 37 °C in the dark for 15 min. Stained wells were washed once with sterile distilled water, which was then added to each well (300 µL). Images were acquired with an inverted Leica SP5 CLSM using a 1.25× dry objective (WD 3.7 mm, Z-resolution 27 µm, up to 24 Z-stacks per well) to visualize an entire well. Samples were kept at the cultivation conditions (37 °C, 5% CO_2_, 90% humidity) with an OkoLabs stage top environmental chamber (Oko Labs, PA, USA). Fluorophore excitation lasers and emission bandwidths were as follows: SYTO^®^ 9 (ex/em 485/498 nm) 488 nm excitation, 495 to 550 nm emission collection; propidium iodide (ex/em 535/617 nm) 561 nm excitation, 595 to 650 nm emission collection. The laser beam scanned the visual field at a frequency of 600 Hz. The wells were captured in snapshots of 1024 × 1024 pixels representing the area of 153.76 mm^2^.

### 4.8. Cytotoxicity Assay

To test the acute cytotoxicity of the compounds, the following cell lines were used: primary human renal tubular epithelial cells (HRTEC; Evercyte, Vienna, Austria); human keratinocytes (HaCat; Thermo Fisher Scientific, Waltham, MA, USA); human dermal fibroblasts (HDF; Merck, Kenilworth, NJ, USA); human epithelial kidney cells (HEK 293; ATCC, Manassas, VI, USA); primary human coronary artery endothelial cells (HCAEC; ATCC, Manassas, VI, USA). The cell lines were maintained in a proper cultivation medium—HaCat, HDF, and Hek 293 in DMEM; HRTEC in VCB; HCAEC in ProxUp. The cytotoxicity experiment was realized according to Tran et al. [41]. Briefly, the cells were counted by the Cellometer Auto T4 (Nexcelom Bioscience, Lawrence, MA, USA), and the cell suspension containing a cell density of 10^5^ cells/mL was split into the 96-well plate, with 100 µL per well. The plates were then incubated for 24 h at 37 °C in a humidified atmosphere of 5% CO_2_. Then, the plates were washed three times with PBS, and the tested natural compounds were added using binary dilution in a proper medium. After 72 h of incubation, the cell viability was tested by resazurin assay. The fluorescence was measured by a SpectraMax i3× microplate reader (San Jose, CA, USA) at a wavelength of 560 nm excitation/590 nm emission.

### 4.9. Statistical Analysis

All MICs measurements were performed in at least two independent experiments, each with three technical replicates. The MICs were calculated as an average of all measured values and represent the minimum concentrations which resulted in at least 80% inhibition of growth (MICPC_80_, MICBC_80_), metabolism (MICBM_80,_ MICMPB_80_) and biofilm formation (MICBF_80_), or resulted in at least 80% reduction in mass of preformed biofilms (MICBR_80_). The significance of the results was verified by *t*-test (*p* = 0.05) using Statistica 13.5.0 (TIBCO Software Inc., Palo Alto, CA, USA).

All collected images were statistically evaluated to quantify biovolume utilizing the surface estimation procedure described in Parker et al. [42]. Briefly, in MATLAB (version R2020a; MathWorks, MA, USA), optimal Beer’s thresholds were calculated for each channel of each collected image by fitting Beer’s Law to the 3D intensity data, and utilizing a Bayesian optimality criterion, the maximum of the posterior probability distribution [43]. Following the image thresholding, biofilm volumes in each channel were calculated as the number of pixels with intensities above Beer’s threshold. The volumes from the treated and control samples were compared to determine the percent reduction in the volumes in the green channel (live cells) and percent increases in volumes in the red channel (membrane-compromised cells). The total biofilm volume was used to calculate an average biofilm thickness for evaluation of statistical significance using the formula below (Equation (2)):

(2) $$\mathrm{biofilm}\;\mathrm{thickness}\;\left(µm\right)=\frac{\mathrm{total}\;\mathrm{biofilm}\;\mathrm{volume}\;}{{\left(12,400\right)}^{2}\;µm}$$

where total biofilm volume is the biofilm volume from bright pixels, and the value ${\left(12,400\right)}^{2}\;µm$ is the field of view of the 1.25× CLSM objective.

In each channel separately, volumes across different treatment types were statistically analyzed by fitting a One-way ANOVA followed by Tukey’s post hoc comparisons. The constant variance and normality assumptions of the ANOVA were assessed by residual plots. The ANOVA was fit using the statistical software R version 4.0.3 [44]. The results were considered to be statistically significant if *p* < 0.05 as mentioned for each experiment.

The cytotoxicity results are expressed as the average IC_50_ ± standard error of the mean (SEM). Values of IC_50_ were obtained by using the online tool Quest Graph IC_50_ Calculator (AAT Bioquest Inc., Sunnyvale, CA, USA). One-way analysis of variance (ANOVA) was used followed by Duncan’s post hoc test (*p* < 0.05) to show the differences between the groups. For ANOVA, the Statistica software (Tibco Software Inc., Palo Alto, CA, USA) was used in version 12.

## 5. Conclusions

In conclusion, the present study evaluated the possible antimicrobial and antibiofilm efficacy of NAC, RLs, and UA against important food-borne pathogens. NAC demonstrated bacterial growth inhibition for both Gram-positive and Gram-negative bacteria, while RLs showed overall lower inhibition, and UA inhibited only the growth of Gram-positive strains. Microscopy investigation revealed that all treatments resulted in cell wall disruption and intracellular component leakage. Furthermore, UA treatment most likely triggered the inhibition of DNA and RNA synthesis, thus resulting in bacterial growth disruption. Even though tested substances did not exhibit promising activity in biofilm removal, the findings in this study indicate the efficacy of NAC in biofilm prevention. Moreover, the in vitro cytotoxicity assay confirmed its relatively low acute cytotoxicity in comparison to RLs and UA. Therefore, it could be potentially used as an additional sanitation strategy and improve biofilm control in the food processing industry. However, further investigations are needed to validate its antibiofilm properties and effective concentration against a broad range of food-borne pathogens.

## Figures and Tables

**Table 1:** Minimal inhibitory concentrations of *N*-Acetyl-l-cysteine (NAC), rhamnolipids (RLs), and usnic acid (UA) for planktonic growth, biofilm formation, and the resulting biofilm metabolic activity. The significance of the results was verified by *t*-test (*p* = 0.05).

Bacterial Strain	NAC (µg/mL)			RLs (µg/mL)			UA (µg/mL)		
	MICPC_80_	MICBF_80_	MICBM_80_	MICPC_80_	MICBF_80_	MICBM_80_	MICPC_80_	MICBF_80_	MICBM_80_
*S. aureus* 816	**3130**	**3130**	**3130**	>1000/49% *	**62.5**	**62.5**	**0.24**	**0.24**	**0.24**
*S. aureus* 1241	**3130**	**12,500**	**6250**	>1000/57% *	**125**	**125**	**0.24**	**0.24**	**0.49**
*L. monocytogenes* 149	**3130**	**6250**	**12,500**	**250**	**62.5**	**62.5**	**0.98**	**0.98**	**1.96**
*L. monocytogenes* 164	**6250**	**6250**	**6250**	>1000/75% *	**125**	**62.5**	**1.96**	**1.96**	**1.96**
*E. coli* 683/17	**3130**	**6250**	**6250**	>1000/29% *	**250**	**500**	>31.3/20% *	>31.3/10% *	>31.3/27% *
*E. coli* 693/17	**6250**	**6250**	**6250**	>1000/23% *	**125**	**250**	>31.3/40% *	>31.3/47% *	>31.3/16% *
*S.* Infantis S13	**6250**	**12,500**	**12,500**	>1000/50% *	**125**	**1000**	>31.3/25% *	>31.3/45% *	>31.3/41% *
*S.* Infantis S59	**6250**	**6250**	**6250**	>1000/39% *	**15.65**	**500**	>31.3/31% *	>31.3/34% *	>31.3/47% *
*S.* Enteritidis ATCC 13076	**12,500**	**12,500**	**12,500**	>1000/33% *	>1000/16% *	**1000**	>31.3/42% *	>31.3/25% *	>31.3/45% *

Tested concentrations: NAC 50,000, 25,000, 12,500, 6250, 3130, 1560, 780, 390, 200, and 100 µg/mL (10 different concentrations); RLs: 1000, 500, 250, 125, 62.5, 31.3, 15.65, 7.83, 3.91, and 1.96 µg/mL (10 different concentrations); UA: 31.3, 15.65, 7.83, 3.91, 1.96, 0.98, 0.49, 0.24, 0.12, and 0.06 µg/mL (10 different concentrations). MICPC_80_—minimum inhibitory concentrations for planktonic growth; MICBF_80_—minimum inhibitory concentrations for biofilm formation; MICBM_80_—minimum inhibitory concentrations for biofilm metabolic activity; bold font marks the efficiency of ≥80%; * marks maximal achieved inhibition.

**Table 2:** Minimal inhibitory concentrations of *N*-Acetyl-l-cysteine (NAC), rhamnolipids (RLs), and usnic acid (UA) inhibiting further growth of biofilm cells, its metabolic activity, and resulting in biofilm reduction. The significance of the results was verified by *t*-test (*p* = 0.05).

Bacterial Strain	NAC (µg/mL)			RLs (µg/mL)			UA (µg/mL)		
	MICBC_80_	MICBR_80_	MICMPB_80_	MICBC_80_	MICBR_80_	MICMPB_80_	MICBC_80_	MICBR_80_	MICMPB_80_
*S. aureus* 816	**3130**	**12,500**	**12,500**	**2000**	**62.5**	**62.5**	**1.96**	>31.3/35% *	>31.3/67% *
*S. aureus* 1241	**6250**	>100,000/31% *	**6250**	**2000**	**1000**	**250**	**3.91**	>31.3/72% *	**3.91**
*L. monocytogenes* 149	**3130**	**100,000**	**6250**	**62.5**	**2000**	**62.5**	**1.96**	>31.3/69% *	**3.91**
*L. monocytogenes* 164	**3130**	>100,000/46% *	**6250**	**250**	**250**	**125**	**1.96**	**1.96**	**1.96**
*E. coli* 683/17	**6250**	>100,000/68% *	**12,500**	**2000**	**2000**	**2000**	>31.3/44% *	>31.3/0% *	>31.3/45% *
*E. coli* 693/17	**3130**	**100,000**	**12,500**	**2000**	**2000**	**2000**	>31.3/61% *	>31.3/0% *	>31.3/20% *
*S.* Infantis S13	**6250**	**12,500**	**12,500**	**2000**	**1000**	**2000**	>31.3/33% *	>31.3/30% *	>31.3/17% *
*S.* Infantis S59	**12,500**	**100,000**	**12,500**	**2000**	**500**	**2000**	>31.3/31% *	>31.3/21% *	>31.3/26% *
*S.* Enteritidis ATCC 13076	**6250**	**12,500**	**12,500**	**2000**	**2000**	**1000**	>31.3/51% *	>31.3/0% *	>31.3/53% *

Tested concentrations: NAC: 100,000, 50,000, 25,000, 12,500, 6250, and 3130 µg/mL (6 different concentrations); RLs: 2000, 1000, 500, 250, 125, and 62.5 µg/mL (6 different concentrations); UA: 31.3, 15.65, 7.83, 3.91, and 1.96 µg/mL (5 different concentrations); MICBC_80_—minimum inhibitory concentrations for further growth of biofilm cells; MICBR_80_—minimum inhibitory concentrations for biofilm reduction; MICMPB_80_—minimum inhibitory concentrations for metabolic activity of preformed biofilm; bold font marks the efficiency of ≥80%; * marks maximal achieved inhibition.

**Table 3:** Biofilm Prevention: Total biofilm thickness for individual organisms with the respective treatment types (*N*-Acetyl-l-cysteine (NAC), rhamnolipids (RLs), and usnic acid (UA)) following 24 h of exposure. Data represent the mean and standard deviation of at least four independent technical replicates.

Bacterial Strain	NAC		RLs		UA		CONTROL
	Thickness (µm) MICBM_80_	Thickness (µm) subCBM80	Thickness (µm) MICBM_80_	Thickness (µm) subCBM80	Thickness (µm) MICBM_80_	Thickness (µm) subCBM80	Thickness (µm)
*S. aureus* 816	**104 ± 67.8** *	**181 ± 57.7** ***	217 ± 38.5	244 ± 28.5	237 ± 41.9	270 ± 21	244 ± 22.4
*S. aureus* 1241	236 ± 50.5	**212 ± 16.1** ***	266 ± 25.5	285 ± 67.4	**367** ** ± 40.5 ** ***	**392** ** ± 21.7 ** **	298 ± 58
*L. monocytogenes* 149	**86.5** ± 2.6 *	238 ± 32	239 ± 49.6	260 ± 33.3	**175** ** ± 79.4 ** ***	268 ± 68.9	243 ± 25.6
*L. monocytogenes* 164	**158** ± 93.2 ***	288 ± 40.6	337 ± 74.1	**348** ** ± 49.6 ** ***	325 ± 70.5	**417** ** ± 40.4 ** *	258 ± 72.9
*E. coli* 683/17	**271** ** ± 50.7 ** *	**229** ** ± 9.9 ** ***	**234** ** ± 30.4 ** ***	**259** ** ± 37.4 ** **	N/A	**269** ** ± 28.1 ** **	162 ± 65.2
*E. coli* 693/17	273 ± 40.8	181 ± 21.9	205 ± 84.5	230 ± 26	N/A	256 ± 12.6	222 ± 34.3
*S.* Enteritidis ATCC 13076	117 ± 41.6	137 ± 69.6	144 ± 37.1	150 ± 10.9	N/A	77.6 ± 39.1	139 ± 64.6

bold font marks statistically significant effect; * marks *p* < 0.001, ** marks *p* < 0.01, *** marks *p* < 0.05; light-colored blue background marks decreased thickness; light-colored orange background marks increased thickness; N/A marks concentration was not applied.

**Table 4:** Biofilm Removal: Total biofilm thickness for individual organisms with the respective treatment types (*N*-Acetyl-l-cysteine (NAC), rhamnolipids (RLs), and usnic acid (UA)) following 20 h of exposure. Data represent the mean and standard deviation of at least four independent technical replicates.

Bacterial Strain	NAC		RLs		UA		CONTROL
	Thickness (µm) MICBM_80_	Thickness (µm) subCBM80	Thickness (µm) MICBM_80_	Thickness (µm) subCBM80	Thickness (µm) MICBM_80_	Thickness (µm) subCBM80	Thickness (µm)
*S. aureus* 816	156 ± 80.4 **	247 ± 64.7	344 ± 87.3	317 ± 33.1	N/A	**152** ± 68.6 **	299 ± 47.5
*S. aureus* 1241	253 ± 44.4	266 ± 37.9	222 ± 23.5	267 ± 26.8	210 ± 32.5	251 ± 15.7	247 ± 58.1
*L. monocytogenes* 149	**338** ± 85.7 ***	**332** ± 51.2 ***	302 ± 41	291 ± 52.3	**317** ± 62.3 ***	276 ± 26.8	237 ± 61.9
*L. monocytogenes* 164	345 ± 62	335 ± 23.1	328 ± 84.2	348 ± 21.5	**440** ± 60.8 **	N/A	285 ± 90.5
*E. coli* 683/17	153 ± 33.3	**128** ** ± 31.6 **	133 ± 49.8	**249** ± 14.9 **	N/A	**285** ± 38.5 **	178 ± 64.8
*E. coli* 693/17	**222** ± 68.8 **	181 ± 33.7	194 ± 7.2	**266** ± 27.1 ***	N/A	**254** ± 81.8 ***	149 ± 9.5
*S.* Enteritidis ATCC 13076	212 ± 16.6	229 ± 54.9	**128** ± 34.3 **	181 ± 10.7	N/A	203 ± 19.8	193 ± 17.7

bold font marks statistically significant effect; * marks *p* < 0.001, ** marks *p* < 0.01, *** marks *p* < 0.05; light-colored blue background marks decreased thickness; light-colored orange background marks increased thickness; N/A marks concentration was not applied.

**Table 5:** Cytotoxicity of natural substances expressed as a concentration that halves the viability of human cells (IC_50_). The data are presented as an average of 3 repetitions with SEM. The significance of the results was verified by ANOVA (Duncan’s post hoc test, *p* < 0.05).

IC_50_ (µg/mL)			
Cell Lines	NAC	RLs	UA
HRTEC	1570 ± 70 ^b^	56.95 ± 0.65 ^d^	3.06 ± 0.17 ^g^
HaCat	4020 ± 470 ^a^	38.74 ± 4.96 ^de^	0.59 ± 0.14 ^h^
HDF	460 ± 20 ^c^	47.64 ± 2.43 ^de^	4.35 ± 0.41 ^f^
HEK 293	1940 ± 250 ^b^	31.48 ± 4.31 ^e^	2.75 ± 0.22 ^g^
HCAEC	1830 ± 90 ^b^	27.50 ± 0.82 ^e^	0.03 ± 0.01 ^i^

Cell lines: primary human renal tubular epithelial cells (HRTEC), human keratinocytes (HaCat), human dermal fibroblasts (HDF), human epithelial kidney cells (HEK 293), and primary human coronary artery endothelial cells (HCAEC). Letters indicate statistically significant groupings. Groups that are statistically significantly different have different letters.

**Table 6:** List of bacterial strains and their origin.

Bacterial Strain	Origin	
*Staphylococcus aureus* 816	Frozen sea fish	Department of Biochemistry and Microbiology, UCT Prague, Czech Republic
*Staphylococcus aureus* 1241	Cow milk	Department of Biochemistry and Microbiology, UCT Prague, Czech Republic
*Listeria monocytogenes* 149	Pork ham	Department of Biochemistry and Microbiology, UCT Prague, Czech Republic
*Listeria monocytogenes* 164	Pork ham	Department of Biochemistry and Microbiology, UCT Prague, Czech Republic
*Escherichia coli* 683/17	Salt bath (cheese industry)	Veterinary Research Institute Brno, Czech Republic
*Escherichia coli* 693/17	Floor (cheese industry)	Veterinary Research Institute Brno, Czech Republic
*Salmonella enterica* Infantis S13	Wastewater treatment plant	Department of Biochemistry and Microbiology, UCT Prague, Czech Republic
*Salmonella enterica* Infantis S59	Frozen chicken meat	Department of Biochemistry and Microbiology, UCT Prague, Czech Republic
*Salmonella enterica* Enteritidis ATCC 13076	Culture collection	American Type Culture Collection, Manassas, VA, USA

## Data Availability

Data are available on request to the corresponding author.

## Supplementary Materials

The following materials are available online at https://www.mdpi.com/article/10.3390/ijms222111307/s1.
