# Supplementary material for: Evaluation of the Antimicrobial Efficacy of N-Acetyl-l-Cysteine, Rhamnolipids, and Usnic Acid—Novel Approaches to Fight Food-Borne Pathogens
Source: Int J Mol Sci. 2021 Oct 20;22(21):11307. doi: 10.3390/ijms222111307 (PMC8583417; doi:10.3390/ijms222111307)
Supplement: Supplementary file 1 [file ijms-22-11307-s001.zip › ijms-1376937-SI.pdf]

Supplementary Materials

Evaluation of The Antimicrobial Efficacy of N-Acetyl-L-Cysteine, Rhamnolipids, and Usnic Acid—Novel Approaches to Fight Food-Borne Pathogens

Ondrej Chlumsky*[1], Heidi J. Smith[2,3], Albert E. Parker[2,4], Kristen Brileya[2], James N. Wilking[2,5], Sabina Purkrtova[1], Hana Michova[1], Pavel Ulbrich[1], Jitka Viktorova[1] and Katerina Demnerova[1]

1 Department of Biochemistry and Microbiology, University of Chemistry and Technology, Technicka 5, 166 28, Prague 6, Czech Republic
2 Center for Biofilm Engineering, Montana State University, Bozeman, MT, 59717, United States
3 Department of Microbiology and Cell Biology, Montana State University, Bozeman, MT, 59717, United States
4 Department of Mathematical Sciences, Montana State University, Bozeman, MT, 59717, United States
5 Chemical and Biological Engineering Department, Montana State University, Bozeman, MT, 59717, United States
* Correspondence: chlumsko@vscht.cz

Table S1. Biofilm Prevention: Reduction/Increase proportion of live cells (Syto9 stained cells) in biofilm volume for individual organisms with the respective treatment types (N-acetyl-L-cysteine (NAC), rhamnolipids (RLs), and usnic acid (UA)) following 24 h exposure, reported as 100 x (1 - (treated abundance)/(control abundance)).

BACTERIAL STRAIN	NAC		RLs		UA	
	**MICBM$_{80}$**	**subCBM$_{80}$**	**MICBM$_{80}$**	**subCBM$_{80}$**	**MICBM$_{80}$**	**subCBM$_{80}$**
S. aureus 816	**66 %**	**30 %**	**24 %**	13 %	11 %	4 %
S. aureus 1241	**37 %**	**39 %**	**38 %**	**28 %**	**28 %**	**35 %**
L. monocytogenes 149	**68 %**	**36 %**	**46 %**	**42 %**	**40%**	8 %
L. monocytogenes 164	**63 %**	25 %	**38 %**	25 %	11 %	18 %
E. coli 683/17	**16 %**	5 %	8 %	3 %	**N/A**	4 %
E. coli 693/17	17 %	**32 %**	6 %	10 %	**N/A**	8 %
S. Enteritidis ATCC 13076	**68 %**	**38 %**	**46 %**	**39 %**	**N/A**	**80 %**

bold font marks statistical significant effect; light-colored blue background marks decreased amount of live cells; light-colored orange background marks increased amount of live cells; N/A marks concentration was not applied

Table S2. Biofilm Prevention: Reduction/Increase of membrane proportion of compromised cells (PI-stained cells) in biofilm volume for individual organisms with the respective treatment types (N-acetyl-L-cysteine (NAC), rhamnolipids (RLs), and usnic acid (UA)) following 24h exposure, reported as 100 x ((treated abundance)/(control abundance) – 1).

BACTERIAL STRAIN	NAC		RLs		UA	
	**$MICBM_{80}$**	**$subCBM_{80}$**	**$MICBM_{80}$**	**$subCBM_{80}$**	**$MICBM_{80}$**	**$subCBM_{80}$**
S. aureus 816	**45 %**	20 %	8 %	18 %	8 %	20 %
S. aureus 1241	1 %	15 %	**26 %**	**27 %**	17 %	**27 %**
L. monocytogenes 149	5 %	**622 %**	**810 %**	**914 %**	188 %	**349 %**
L. monocytogenes 164	**327 %**	**564 %**	**1053 %**	**930 %**	**584 %**	**700 %**
E. coli 683/17	**151 %**	**118 %**	**130 %**	**153 %**	**N/A**	**181 %**
E. coli 693/17	28 %	7 %	9 %	15 %	**N/A**	35 %
S. Enteritidis ATCC 13076	**168 %**	126 %	**179 %**	**171 %**	**N/A**	82 %

bold font marks statistical significant effect; light-colored blue background marks decreased amount of membrane-compromised cells; light-colored orange background marks increased amount of membrane-compromised cells; N/A marks concentration was not applied

Table S3. Biofilm Prevention: Total biofilm proportion reduction/increase (Syto9+ PI-stained cells) for individual organisms with the respective treatment types (N-acetyl-L-cysteine (NAC), rhamnolipids (RLs), and usnic acid (UA)) following 24h exposure, reported as 100 x (1 - (treated abundance)/(control abundance)).

BACTERIAL STRAIN	NAC		RLs		UA	
	**MICBM$_{80}$**	**subCBM$_{80}$**	**MICBM$_{80}$**	**subCBM$_{80}$**	**MICBM$_{80}$**	**subCBM$_{80}$**
S. aureus 816	**57 %**	**26 %**	11 %	0 %	3 %	11 %
S. aureus 1241	21 %	**29 %**	11 %	4 %	**23 %**	**31 %**
L. monocytogenes 149	**64 %**	2 %	2 %	7 %	**28 %**	10 %
L. monocytogenes 164	**39 %**	12 %	30 %	**35 %**	26 %	**61 %**
E. coli 683/17	**68 %**	**42 %**	**45 %**	**61 %**	**N/A**	**66 %**
E. coli 693/17	23 %	18 %	8 %	4 %	**N/A**	15 %
S. Enteritidis ATCC 13076	16 %	1 %	4 %	8 %	**N/A**	44 %

bold font marks statistical significant reduction; light-colored blue background marks decreased total biofilm volume and light-colored orange background marks increased total biofilm volume when compared to the control; N/A marks concentration was not applied

Table S4. Biofilm Prevention: Statistically significant differences (p values) in proportion of membrane-compromised cells from the control.

BACTERIAL STRAIN	NAC		RLs		UA	
	**$MICBM_{80}$**	**$subCBM_{80}$**	**$MICBM_{80}$**	**$subCBM_{80}$**	**$MICBM_{80}$**	**$subCBM_{80}$**
S. aureus 816	0.22810	0.47860	**0.00021**	**< 0.00001**	**0.03298**	**0.00025**
S. aureus 1241	**0.00952**	**0.00021**	**< 0.00001**	**0.00001**	0.13160	0.10352
L. monocytogenes 149	**0.03947**	**0.00012**	**0.00004**	**0.00003**	**0.04172**	0.05595
L. monocytogenes 164	**0.00871**	0.12062	0.34000	0.08747	0. 07528	0.19371
E. coli 683/17	**0.04633**	**0.03823**	**0.03082**	**0.03369**	N/A	**0.02145**
E. coli 693/17	**0.01559**	**0.00297**	**0.00054**	**0.00554**	N/A	**0.00001**
S. Enteritidis ATCC 13076	**0.00217**	**0.00712**	**0.00444**	**0.00334**	N/A	**0.00424**

bold font marks statistical significant effect; N/A marks concentration was not applied

Table S5. Biofilm Removal: Reduction/Increase proportion of live cells (Syto9 stained cells) in biofilm volume for individual organisms with the respective treatment types (N-acetyl-L-cysteine (NAC), rhamnolipids (RLs), and usnic acid (UA)) following 20 h exposure, reported as 100 x (1 - (treated abundance)/(control abundance)).

BACTERIAL STRAIN	NAC		RLs		UA	
	**$MICMPB_{80}$**	**$subCMPB_{80}$**	**$MICMPB_{80}$**	**$subCMPB_{80}$**	**$MICMPB_{80}$**	**$subCMPB_{80}$**
S. aureus 816	**67 %**	13 %	8 %	9 %	**N/A**	**66 %**
S. aureus 1241	5 %	1 %	**30 %**	13 %	**27 %**	9 %
L. monocytogenes 149	2 %	5 %	**38 %**	**39 %**	11 %	17 %
L. monocytogenes 164	4 %	6 %	**33 %**	**32 %**	**25 %**	**N/A**
E. coli 683/17	3 %	**30 %**	**31 %**	6 %	**N/A**	7 %
E. coli 693/17	**26 %**	10 %	13 %	11 %	**N/A**	4 %
S. Enteritidis ATCC 13076	3 %	**13 %**	**16 %**	11 %	**N/A**	1 %

bold font marks statistical significant effect; light-colored blue background marks decreased amount of live cells; light-colored orange background marks increased amount of live cells; N/A marks concentration was not applied

Table S6. Biofilm Removal: Reduction/Increase proportion of membrane-compromised cells (PI-stained cells) in biofilm volume for individual organisms with the respective treatment types (N-acetyl-L-cysteine (NAC), rhamnolipids (RLs), and usnic acid (UA)) following 20 h exposure, reported as 100 x ((treated abundance)/(control abundance) – 1).

BACTERIAL STRAIN	NAC		RLs		UA	
	**MICMPB80**	**subCMPB80**	**MICMPB80**	**subCMPB80**	**MICMPB80**	**subCMPB80**
S. aureus 816	26 %	22 %	24 %	3 %	**N/A**	31 %
S. aureus 1241	14 %	20 %	23 %	**42 %**	4 %	19 %
L. monocytogenes 149	**327 %**	**354 %**	**487 %**	**451 %**	**197 %**	**248 %**
L. monocytogenes 164	**99 %**	**90 %**	**162 %**	**188 %**	**145 %**	**N/A**
E. coli 683/17	53 %	20 %	3 %	**210 %**	**N/A**	**261 %**
E. coli 693/17	**250 %**	**310 %**	**421 %**	**888 %**	**N/A**	**742 %**
S. Enteritidis ATCC 13076	19 %	28 %	**58 %**	0 %	**N/A**	14 %

bold font marks statistical significant effect; light-colored blue background marks decreased amount of membrane-compromised cells; light-colored orange background marks increased amount of membrane-compromised cells; N/A marks concentration was not applied

Table S7. Biofilm Removal: Total biofilm volume reduction/increase (Syto9+ PI-stained cells) for individual organisms with the respective treatment types (N-acetyl-L-cysteine (NAC), rhamnolipids (RLs), and usnic acid (UA)) following 20 h exposure, reported as 100 x (1 - (treated abundance)/(control abundance)).

BACTERIAL STRAIN	NAC		RLs		UA	
	MICMPB$_{80}$	subCMPB$_{80}$	MICMPB$_{80}$	subCMPB$_{80}$	MICMPB$_{80}$	subCMPB$_{80}$
S. aureus 816	**48 %**	17 %	15 %	6 %	**N/A**	**49 %**
S. aureus 1241	3 %	8 %	10 %	8 %	15 %	2 %
L. monocytogenes 149	**43 %**	**40 %**	28 %	23 %	**34 %**	16 %
L. monocytogenes 164	21 %	18 %	15 %	22 %	**55 %**	**N/A**
E. coli 683/17	14 %	**28 %**	25 %	**40 %**	**N/A**	**60 %**
E. coli 693/17	**49 %**	22 %	31 %	**78 %**	**N/A**	**70 %**
S. Enteritidis ATCC 13076	10 %	19 %	**33 %**	6 %	**N/A**	5 %

bold font marks statistical significant reduction; light-colored blue background marks decreased total biofilm volume and light-colored orange background marks increased total biofilm volume when compared to the control; N/A marks concentration was not applied

Table S8. Biofilm Removal: Statistically significant differences (p values) in proportion of membrane-compromised cells from the control.

BACTERIAL STRAIN	NAC		RLs		UA	
	**$MICMPB_{80}$**	**$subCMPB_{80}$**	**$MICMPB_{80}$**	**$subCMPB_{80}$**	**$MICMPB_{80}$**	**$subCMPB_{80}$**
S. aureus 816	**0.013521**	0.324236	0.068309	0.207037	N/A	**0.007789**
S. aureus 1241	0.233135	0.239613	0.065399	0.081904	0.130569	0.179255
L. monocytogenes 149	**0.002262**	**0.001121**	**0.000099**	**0.000149**	**0.044209**	**0.001854**
L. monocytogenes 164	**0.04163**	**0.046919**	**0.005802**	**0.004356**	0.059296	N/A
E. coli 683/17	0.295934	0.375317	0.263687	**0.014672**	N/A	**0.013435**
E. coli 693/17	0.075467	**0.004592**	**< 0.00001**	**< 0.00001**	N/A	**< 0.00001**
S. Enteritidis ATCC 13076	0.206194	0.50000	**0.026407**	0.381482	N/A	0.174209

bold font marks statistical significant effect; N/A marks concentration was not applied
